# CAREGIVER SUBGROUP ANALYSES FROM AN EMBEDDED DEMENTIA CARE EFFECTIVENESS TRIAL

**DOI:** 10.1093/geroni/igac059.346

**Published:** 2022-12-20

**Authors:** Richard Fortinsky, Dorothy Wakefield, Lily Zhong, James Grady, Catherine Piersol, Julie Robison, Laura Gitlin

**Affiliations:** University of Connecticut Center on Aging, Farmington, Connecticut, United States; University of Connecticut, Farmington, Connecticut, United States; University of Connecticut, Farmington, Connecticut, United States; University of Connecticut, Farmington, Connecticut, United States; Jefferson College of Rehabilitation Sciences, Philadelphia, Pennsylvania, United States; UConn Health, Center on Aging, Farmington, Connecticut, United States; Drexel University, Philadelphia, Pennsylvania, United States

## Abstract

Efficacious dementia care interventions for family/informal caregivers are increasingly being tested for effectiveness in “real world” service delivery settings. We conducted an effectiveness trial in which we embedded the Care of Persons with Dementia in their Environments (COPE) program into Connecticut’s publicly-funded Home and Community-Based Services program. COPE is designed to build dementia management skills in caregivers of people with dementia in the home setting. In published results from this trial with the full study cohort, caregivers who received COPE, compared to controls, experienced improved well-being due to the dementia management skills they learned (perceived well-being), but they did not experience reduced levels of distress due to dementia-related behavioral and psychological symptoms (distress). For this presentation, we determined COPE effects for selected caregiver subgroups on perceived well-being and distress. Regarding caregiver sex, we found that COPE effects were statistically significantly positive on both outcomes for females (both p<.05) but on neither outcome for males. Controlling for sex, we also found positive COPE effects on both outcomes for daughters (both p=.03) but on neither outcome for spouses or sons. For perceived well-being, we found positive COPE effects for White (p<.001) but not for Black caregivers. For distress, we found positive COPE effects for caregivers living apart (p=0.03) but not for those living together with people with dementia. Findings suggest that male, Black, and co-residing caregivers may need more support from COPE, and more broadly demonstrate the value of subgroup analyses in offering greater precision when embedding nonpharmacological interventions in effectiveness trials.

